# PIWI-Interacting RNAs in Cardiovascular Disease: From Epigenetic Regulators to Clinical Biomarkers and Therapeutic Targets

**DOI:** 10.31083/RCM48753

**Published:** 2026-07-20

**Authors:** Xingxing Wang, Chang Liu, Xiaodong Wang, Jinyu Pan, Wenyuan Ding, Xiuqing Tian

**Affiliations:** ^1^Department of Cardiology, The First Affiliated Hospital of Shandong First Medical University & Shandong Provincial Qianfoshan Hospital, 250000 Jinan, Shandong, China; ^2^Department of Cardiology, Shandong First Medical University & Shandong Academy of Medical Sciences, 250000 Jinan, Shandong, China; ^3^College of Life Sciences and Technology, Shandong Second Medical University, 261000 Weifang, Shandong, China

**Keywords:** cardiovascular diseases, PIWI-interacting RNAs (piRNAs), biomarkers, heart failure

## Abstract

PIWI-interacting RNAs (piRNAs) are a class of non-coding RNAs approximately 24–32 nucleotides (nt) in length. They are well known for silencing transposons and maintaining genomic integrity during germ cell development. Recent studies have detected specific piRNAs in cardiovascular-relevant tissues, including myocardial tissue, circulating samples, and distinct cardiac cell populations, as well as in various disease settings. However, the biogenesis and functions of somatic piRNA/P-element-induced wimpy testis proteins (PIWI) signaling in mammalian cardiovascular tissues remain incompletely understood. The biological functions of piRNAs in cardiac physiology and the pathogenesis of various diseases are still largely unexplored. In this review, we summarize piRNA expression patterns, underlying mechanisms, and potential clinical significance in cardiovascular diseases, providing insights that may facilitate the development of targeted molecular therapies.

## 1. Introduction

### 1.1 Background: Cardiovascular Disease and piRNA Biology

Cardiovascular diseases (CVDs) are a leading cause of death worldwide and encompass disorders involving structural and functional abnormalities of the heart and blood vessels. According to the World Health Organization, an estimated 19.8 million people died from CVDs in 2022 [1]. Recent projection analyses further suggest that global CVD prevalence may increase by 90.0% between 2025 and 2050, with cardiovascular deaths reaching 35.6 million by 2050 [2]. CVDs encompass a broad range of disorders affecting the heart and blood vessels, including myocardial infarction, cardiomyopathy, heart failure, and atherosclerotic vascular disease [1]. Established risk factors for CVDs include hyperlipidemia, hypertension, and obesity. Despite substantial advances in clinical diagnostics, more sensitive and disease-specific molecular biomarkers for early detection, risk stratification, and prognostic evaluation remain needed in cardiovascular disease [3]. Therefore, new diagnostic biomarkers and therapeutic interventions are essential to minimize the risk of CVD. Over the past decade, a large body of evidence has demonstrated that noncoding RNAs (ncRNAs) play key regulatory roles in various organs. Small ncRNAs and long ncRNAs are the two major classes of ncRNAs. Among the small ncRNAs, there are three types of RNAs in eukaryotes: microRNAs (miRNAs), PIWI-interacting RNAs (piRNAs) and small interfering RNAs (siRNAs) [4]. The piRNAs are small RNAs defined by their ability to specifically bind PIWI proteins [5]. piRNAs have 2′-O-methylated 3′ termini and usually exhibit a 5′ uridine bias [6,7,8,9]. piRNAs were originally thought to exist only in germ cells [7,10]. However, recent studies have shown that they are also detected in neural, immune, cardiovascular, and tumor-related contexts [11,12,13,14]. Derived from single-stranded precursors via an RNase III–independent pathway, piRNAs help preserve genome integrity by epigenetically repressing transposons through DNA methylation and by modulating gene expression *in vivo*, thereby contributing to the development of diverse diseases [6,15].

### 1.2 Review Scope and Literature Search Strategy

This article is intended as a narrative review, rather than a formal systematic review. While the title broadly refers to cardiovascular disease, this review focuses on four representative conditions: myocardial infarction (MI), hypertrophic cardiomyopathy (HCM), pulmonary arterial hypertension (PAH) and heart failure (HF). These conditions were selected because they represent major pathological categories within the cardiovascular disease spectrum, and there is currently the most substantial and translationally relevant evidence regarding piRNA involvement in them.

To improve transparency, a literature search was performed in PubMed for articles published up to December 2025 using the following combinations of keywords: ‘piRNA’, ‘PIWI-interacting RNA’, ‘cardiovascular disease’, ‘myocardial infarction’, ‘hypertrophic cardiomyopathy’, ‘pulmonary arterial hypertension’, ‘heart failure’, ‘biomarker’, ‘epigenetics’, and ‘therapeutic target’. Reference lists of relevant reviews and original studies were also manually screened to identify additional articles. As this review was designed as a narrative synthesis, formal systematic screening procedures were not applied. Priority was instead given to peer-reviewed studies that were directly relevant to cardiovascular piRNA biology, provided mechanistic insight, and had potential clinical or translational significance.

## 2. Overview of piRNAs

### 2.1 piRNA Biogenesis

**Fig. 1.**
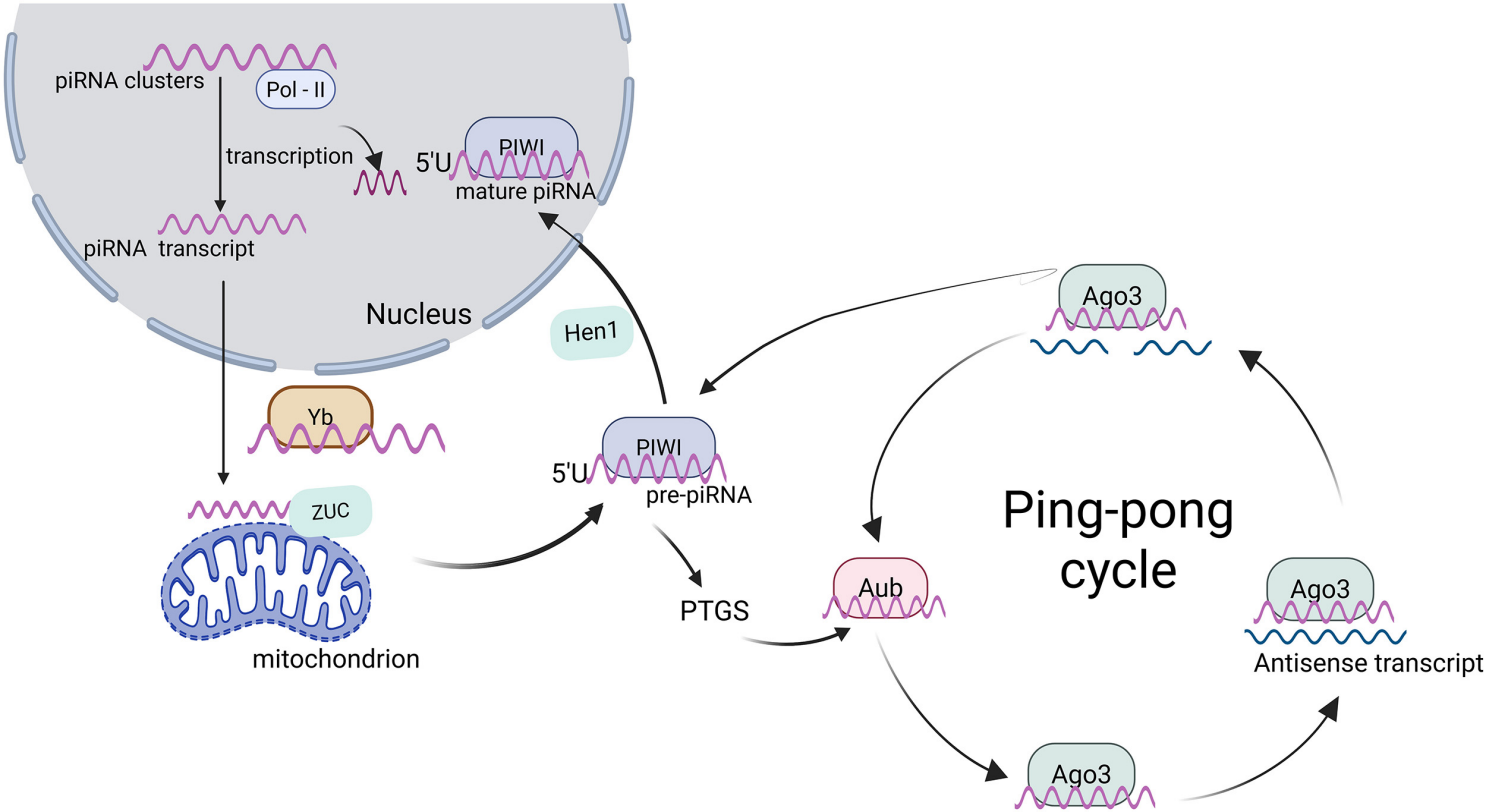
**piRNA biogenesis**. Primary and secondary piRNA biogenesis pathways. piRNA, PIWI-interacting RNAs. Created in BioRender (https://www.biorender.com/).

piRNAs are processed from piRNA precursors. Much of the canonical mechanistic framework for piRNA biogenesis is derived from Drosophila germline and ovarian somatic models. Therefore, organism-specific structures such as the Yb body should be interpreted as Drosophila-specific unless otherwise stated, rather than as established processing compartments in mammalian cardiovascular tissues [16]. Like other small RNAs, piRNAs bind Argonaute-family proteins; however, their biogenesis is Dicer-independent and instead relies on association with PIWI proteins. In canonical model systems, internal ribonucleoprotein granules and the mitochondrial outer membrane have been implicated as important sites of piRNA production, and several core features of piRNA biogenesis appear to be conserved across species. However, the corresponding subcellular organization in mammalian cardiovascular somatic tissues remains incompletely defined [17,18]. In canonical model systems, piRNA biogenesis is generally described as involving primary and secondary pathways, although equivalent compartmental organization in mammalian cardiovascular somatic cells has not been fully established [19] (Fig. 1). In the Drosophila ovary environment, primary biogenesis functions in both somatic follicles and germ cells, whereas secondary biogenesis—known as the “ping-pong” cycle—is germ cell specific. piRNA biosynthesis begins with the transcription of single-stranded piRNA clusters by RNA polymerase II [20]. In Drosophila somatic cells, Yb-body-associated precursor transcripts are delivered to the mitochondrial surface, where they are processed by Zucchini (Zuc), a mitochondria-associated endoribonuclease, to generate the 5′ ends of pre-piRNAs [21,22]. In mammals, although core principles such as Dicer-independent processing, PIWI association, and mitochondrial-linked precursor maturation appear broadly conserved, the precise subcellular organization of somatic piRNA biogenesis remains less well defined, particularly in cardiovascular tissues [4]. The resulting pre-piRNAs are subsequently 3′-end trimmed to the mature length and then 2′-O-methylated at their 3′ termini by Hen1 to form mature piRNAs [23,24]. This process constitutes primary piRNA biogenesis [22,25]. In germ cells, secondary piRNA biogenesis proceeds through the ping-pong cycle, in which Aub- and Ago3-containing complexes mediate reciprocal slicer-dependent cleavage of complementary transposon transcripts, thereby generating and amplifying secondary piRNAs [26]. For a cardiovascular medicine readership, it is important to note that the detailed compartmental organization of primary and secondary piRNA biogenesis has been most clearly defined in Drosophila and germline systems, whereas equivalent somatic biogenesis pathways in mammalian cardiovascular tissues remain incompletely characterized (Fig. 1).

### 2.2 Function of piRNAs and Related Mechanisms

piRNAs exert their functions by binding PIWI-clade Argonaute proteins to form piRNA-induced silencing complexes (piRISCs), which mediate transcriptional and post-transcriptional silencing of transposable elements and other targets [22,27,28]. In slicer-competent PIWI proteins, these complexes recognize complementary target RNAs and mediate endonucleolytic cleavage of target transcripts [22,29]. PIWI proteins function in both germline and somatic contexts, although their expression patterns and family composition vary among species [27]. In humans, four PIWI proteins (PIWIL1–4) have been identified, whereas mice and Drosophila each possess three PIWI homologs [30,31,32]. Overall, core features of the piRNA/PIWI pathway appear to be evolutionarily conserved. However, most mechanistic insights into piRNA/PIWI function derive from germline or Drosophila models. In mammalian cardiovascular tissues, current evidence is stronger for disease-associated expression changes and functional associations of selected piRNAs than for a fully resolved somatic piRNA biogenesis and PIWI-effector framework [33]. PIWI proteins contain three conserved domains: PAZ, MID, and PIWI. The PAZ domain binds the 3′ end of the guide piRNA, whereas the MID domain anchors the 5′ phosphate of the guide [34,35]. The PIWI domain contains the conserved slicer catalytic center and, guided by base pairing between the bound piRNA and its target transcript, mediates endonucleolytic cleavage of complementary target RNA during post-transcriptional silencing and ping-pong amplification [22,36]. Accordingly, domain-level mechanistic models of PIWI slicer activity are broadly informative, but direct extrapolation of canonical germline or Drosophila mechanisms to mammalian cardiovascular cells should be made cautiously.

piRNAs are involved in epigenetic regulation mainly through histone modification and DNA methylation/demethylation known as transcriptional gene silencing (TGS) [37,38]. Currently, two pathways of piRNA-induced DNA methylation have been identified. The first pathway involves direct modification of transposon DNA by the piRNA/PIWI complex. The second pathway involves the piRNA/PIWI complex regulating the expression of DNA methyltransferase (DNMT) to modify transposon DNA [39]. piRNA achieves transposon silencing and genome stability maintenance by modulating histone modifications (especially H3K4me2) [40]. Recent studies have shown that three complexes, including the small ubiquitin-like modifier (SUMO) and SUMO E3 ligases Su(var)2-10, piRNA/PIWI, and SetDB1/Wde, work closely together to induce transcriptional silencing in Drosophila [41]. The role of piRNA-mediated cellular regulation in disease development is increasingly recognized. piRNAs have been explored as potential biomarkers for different diseases, including cardiovascular diseases. Because direct mechanistic evidence in cardiovascular tissues remains relatively limited, selected findings from non-cardiovascular diseases are mentioned here only to illustrate broader piRNA/PIWI-related functions. In cardiovascular disease, current evidence has mainly focused on disease-associated expression changes and biomarker potential. For example, altered serum piRNA profiles have been reported in patients with acute myocardial infarction [42]. Differential expression of piRNAs has been reported in Alzheimer’s disease, suggesting possible diagnostic relevance [43]. piRNA/PIWI-associated regulatory axes have also been described in human cancers, including piR-932/PIWIL2 in breast cancer and piR-54265 in colorectal adenocarcinoma [44,45]. These observations support the broader functional relevance of piRNAs while highlighting the need for more direct mechanistic studies in cardiovascular contexts.

### 2.3 Relevance to Mammalian Cardiovascular Tissues

For mammalian cardiovascular tissues, current evidence mainly supports the presence and disease-associated dysregulation of selected piRNAs in clinically relevant settings, including myocardial tissue, circulating samples, and specific cardiac cell populations [13,33,42]. However, the upstream biogenesis machinery, subcellular processing architecture, and cell-type-specific PIWI dependency of somatic piRNA signaling in mammalian cardiovascular tissues remain incompletely defined. In particular, a mammalian cardiovascular subcellular processing compartment directly analogous to the Drosophila Yb body has not yet been clearly defined in the current literature [17,33]. Therefore, mechanistic extrapolation from canonical Drosophila models should be interpreted with caution, and most current cardiovascular studies should be viewed as functional or association-based rather than as definitive maps of somatic piRNA biogenesis [33].

## 3. piRNAs in CVDs

To improve consistency and interpretability, the following disease-focused sections are organized according to three aspects: (i) discovery and validation evidence, (ii) mechanistic evidence, and (iii) translational relevance and critical appraisal. Because many currently reported cardiovascular piRNAs are derived from exploratory or small-scale studies, the available evidence should be interpreted cautiously, particularly with respect to cohort size, independent replication, sample source, sequencing/annotation pipelines, normalization methods, and the possibility of annotation ambiguity in somatic tissues. In non-gonadal somatic tissues, some reported “piRNAs” may represent fragments derived from other small noncoding RNAs rather than bona fide PIWI-interacting RNAs, which remains an important methodological consideration in cardiovascular studies. The major piRNAs dysregulated in myocardial infarction, hypertrophic cardiomyopathy, pulmonary arterial hypertension, and heart failure are summarized in Table 1 (Ref. [13,37,42,46,47,48,49,50,51]).

**Table 1.** **Major piRNAs dysregulated in cardiovascular diseases and their reported functions/mechanisms**.

piRNA	Expression	Sample	Function	Type of diseases	References
hsa-piR-9010	Up	Serum	Candidate biomarker for MI (exploratory)	MI	[42]
hsa-piR-28646	Up	Serum	Candidate biomarker for MI (exploratory); associated with inflammatory and remodeling pathways	MI	[42]
hsa-piR-23619	Up	Serum	Candidate biomarker for MI (exploratory); associated with inflammatory signaling	MI	[42]
DQ542443 (HAAPIR)	Up	Cardiomyocytes/MI model	Promotes cardiomyocyte apoptosis in MI (preclinical)	MI	[46]
DQ691316 (HNEAP)	Up	Cardiomyocytes/MI model	Promotes cardiomyocyte necroptosis in MI (preclinical)	MI	[47]
CHAPIR (piR-141981/DQ726659)	Up	Hypertrophied cardiomyocytes/hypertrophic heart tissue	Mechanistic regulator of pathological hypertrophy in HCM (preclinical)	HCM	[48]
DQ695228	Up	Hypertrophied cardiomyocytes/hypertrophic heart tissue	HCM-associated piRNA candidate (discovery-stage)	HCM	[48]
DQ545263	Down	Hypertrophied cardiomyocytes/hypertrophic heart tissue	HCM-associated piRNA candidate (discovery-stage)	HCM	[48]
DQ593039	Up	Extracellular vesicles	Candidate biomarker for PH-spectrum disease (EV-based exploratory); PAH specificity uncertain	PH spectrum/PAH specificity uncertain	[49]
piR-63076	Up	Pulmonary arterial smooth muscle cells	Promotes pulmonary vascular remodeling in PAH (preclinical)	PAH	[37]
hsa-piR-020009	Down	Serum exosomes	Candidate biomarker for HF (exploratory)	HF	[50]
hsa-piR-006426	Down	Serum exosomes	Candidate biomarker for HF (exploratory)	HF	[50]
piRNA-6426	Down	Cardiomyocytes/HF model	Protective regulator in HF (preclinical)	HF	[51]
piRNA-000691 (CFRPi)	Up	Cardiac fibroblasts	Promotes cardiac fibrosis in HF (preclinical)	HF	[13]

**Note:** piRNA nomenclature varies across studies and databases. In this manuscript, piRNA names are retained according to the original cited studies unless a confirmed alias relationship is available. Therefore, identifiers such as “hsa-piR-006426” and “piRNA-6426” are presented separately because no confirmed alias mapping could be established. **Abbreviations:** HCM, hypertrophic cardiomyopathy; MI, myocardial infarction; PAH, pulmonary arterial hypertension; PH, pulmonary hypertension; EV, extracellular vesicles; HF, heart failure.

### 3.1 Myocardial Infarction

#### 3.1.1 Discovery and Validation Evidence

Myocardial infarction (MI) remains a major cause of cardiomyocyte death and adverse cardiac remodeling, and there is continued interest in identifying novel molecular biomarkers beyond conventional serum markers. In a serum-based discovery study, Huang et al. [42] performed high-throughput sequencing and bioinformatic analysis in patients with acute MI and healthy controls and reported 195 upregulated and 13 downregulated piRNAs in the acute MI group. Among these, hsa-piR-9010, hsa-piR-28646, and hsa-piR-23619 showed the most prominent differential expression and should currently be regarded as exploratory circulating candidates identified at the discovery stage, rather than as independently validated biomarkers [42].

#### 3.1.2 Mechanistic Evidence

**Fig. 2.**
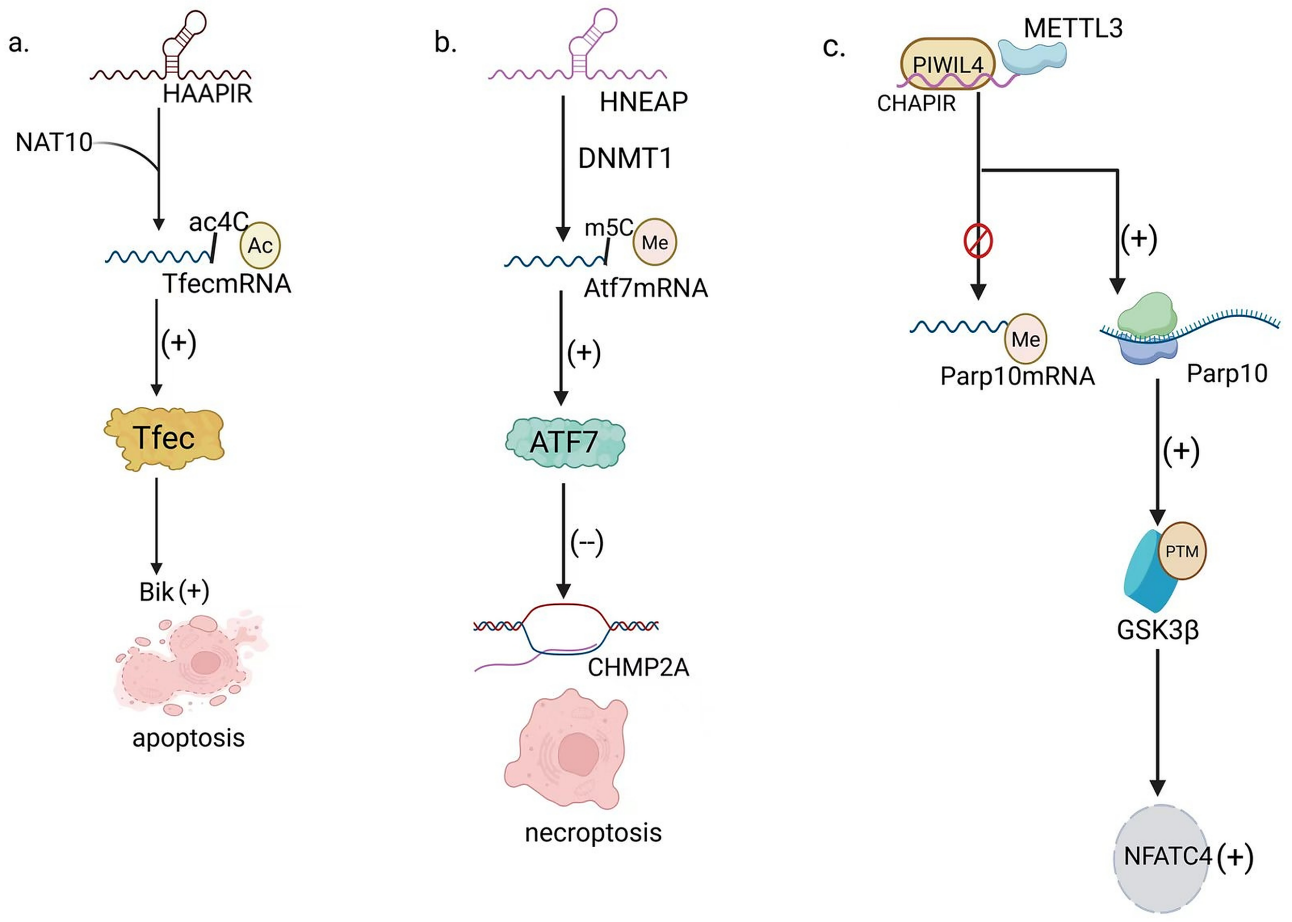
**Mechanistic involvement of piRNAs in distinct cardiovascular diseases (CVDs)**. (a) HAAPIR has been shown to aggravate myocardial injury by promoting cardiomyocyte apoptosis. Mechanistically, HAAPIR enhances NAT10-mediated ac4C modification of *Tfec *mRNA, thereby increasing *Bik* transcription. (b) HNEAP has been reported to participate in necrosis-related cardiac injury in ischemic heart disease through regulation of the m5C methylation of *Atf7* mRNA via interaction with DNMT1. (c) In hypertrophic cardiomyopathy (HCM), the CHAPIR–PIWIL4 complex has been demonstrated to modulate Parp10 expression by suppressing METTL3-dependent m6A modification. Created in BioRender (https://www.biorender.com/).

In the current MI literature, several candidate piRNAs were initially identified through serum-based discovery profiling rather than direct functional experiments. In particular, hsa-piR-9010, hsa-piR-28646, and hsa-piR-23619 were reported as differentially expressed circulating candidates in sequencing datasets from patients with acute myocardial infarction [42]. At this stage, these findings should be interpreted primarily as observed associations in exploratory profiling studies. For hsa-piR-28646 and hsa-piR-23619, the currently available literature supports only limited pathway-level interpretation. Specifically, hsa-piR-28646 has been discussed in relation to Wnt/β-catenin-related signaling, whereas hsa-piR-23619 has been discussed in relation to TNF-related inflammatory signaling in the post-MI setting [42,52,53]. However, these links are better interpreted as putative or inferred pathway associations, rather than as directly validated cardiovascular mechanisms or confirmed target-specific interactions. By contrast, stronger direct preclinical mechanistic evidence is available for heart-apoptosis-associated piRNA (HAAPIR; DQ542443), which has been experimentally shown to promote cardiomyocyte apoptosis by enhancing NAT10-mediated ac4C acetylation of *Tfec* mRNA, thereby increasing *Bik* expression and mitochondrial apoptosis (Fig. 2a) [46]. Similarly, HNEAP (DQ691316) has been reported to promote cardiomyocyte necroptosis by suppressing DNMT1-mediated m5C methylation of *Atf7* mRNA, leading to reduced CHMP2A expression (Fig. 2b) [47].

#### 3.1.3 Translational Relevance and Critical Appraisal

Taken together, MI-associated piRNAs are currently best regarded as candidate circulating biomarkers and preclinical mechanistic regulators rather than clinically validated diagnostic tools or therapeutic targets. Most reported findings remain based on exploratory profiling studies or limited preclinical models, and external validation in larger independent cohorts is still lacking. In addition, cross-study comparability is limited by inconsistent reporting of sequencing pipelines, annotation criteria, and normalization methods. Because these studies are performed in somatic tissues and circulating samples, an additional interpretive concern is that some reported “piRNAs” may represent other small-RNA fragments or annotation artifacts rather than bona fide piRNAs. Accordingly, mechanistic interpretation in MI should distinguish carefully between differential-expression observations, pathway-level inference, and directly validated molecular mechanisms, as these represent different levels of evidentiary support.

### 3.2 Hypertrophic Cardiomyopathy

#### 3.2.1 Discovery and Validation Evidence

Current evidence linking piRNAs to hypertrophic cardiomyopathy (HCM) is derived primarily from experimental transcriptomic analyses rather than from large clinical validation cohorts. By comparing RNA-sequencing data from cardiomyocytes of healthy and hypertrophied hearts, Gao et al. [48] reported increased overall piRNA reads in hypertrophied hearts and identified three differentially expressed candidates—DQ726659, DQ695228, and DQ545263—that were associated with increased expression of the hypertrophic marker β-MHC. These findings support the possibility that piRNA dysregulation accompanies pathological cardiac hypertrophy, but they should currently be regarded as preclinical discovery-stage evidence.

#### 3.2.2 Mechanistic Evidence

It is important to distinguish between discovery-stage differential expression signals and experimentally validated direct mechanisms in the current HCM literature. Although several HCM-associated piRNAs have been identified through transcriptomic analyses, the strongest mechanistic evidence is available for CHAPIR (piR-141981/DQ726659). CHAPIR is highly expressed in hypertrophic cardiomyocytes, and CHAPIR deficiency attenuates cardiac hypertrophy and improves cardiac function [48]. Mechanistically, the CHAPIR–PIWIL4 complex interacts with METTL3 to inhibit m6A methylation of *Parp10* mRNA, thereby increasing PARP10 expression [48,54,55]. Increased PARP10 promotes mono-ADP-ribosylation of GSK3β, suppresses its kinase activity, and facilitates nuclear accumulation of NFATC4, ultimately promoting pathological hypertrophy (Fig. 2c) [48].

#### 3.2.3 Translational Relevance and Critical Appraisal

At present, HCM-associated piRNAs should be considered promising preclinical regulators rather than clinically validated biomarkers. Although CHAPIR represents one of the most mechanistically mature examples in the cardiovascular piRNA field, the available evidence remains dominated by experimental models, and large independent clinical validation datasets are still lacking. Additional limitations include uncertain cross-platform reproducibility, limited annotation standardization, and the need to determine whether candidate transcripts can be robustly measured in clinically relevant human samples before translational application can be considered.

### 3.3 Pulmonary Arterial Hypertension

#### 3.3.1 Discovery and Validation Evidence

Pulmonary arterial hypertension (PAH), corresponding to Group 1 pulmonary hypertension (PH), is characterized by progressive pulmonary vascular remodeling, increased pulmonary vascular resistance, and eventual right ventricular dysfunction. One of the main observations from the current literature comes from profiling small RNAs associated with extracellular vesicles in patients with pulmonary hypertension, rather than in a rigorously restricted Group 1 pulmonary arterial hypertension (PAH)-only cohort. Lipps et al. [49] reported that extracellular vesicle-derived small non-coding RNAs differ between patients with pulmonary hypertension and healthy controls, supporting the concept that circulating vesicle-associated RNA signatures may have biomarker potential. Within this broader PH profiling context, DQ593039 has been described as an upregulated candidate associated with pulmonary hypertension; however, its value as a PAH-specific biomarker remains uncertain and will require formal validation in well-defined Group 1 PAH cohorts [49].

#### 3.3.2 Mechanistic Evidence

**Fig. 3.**
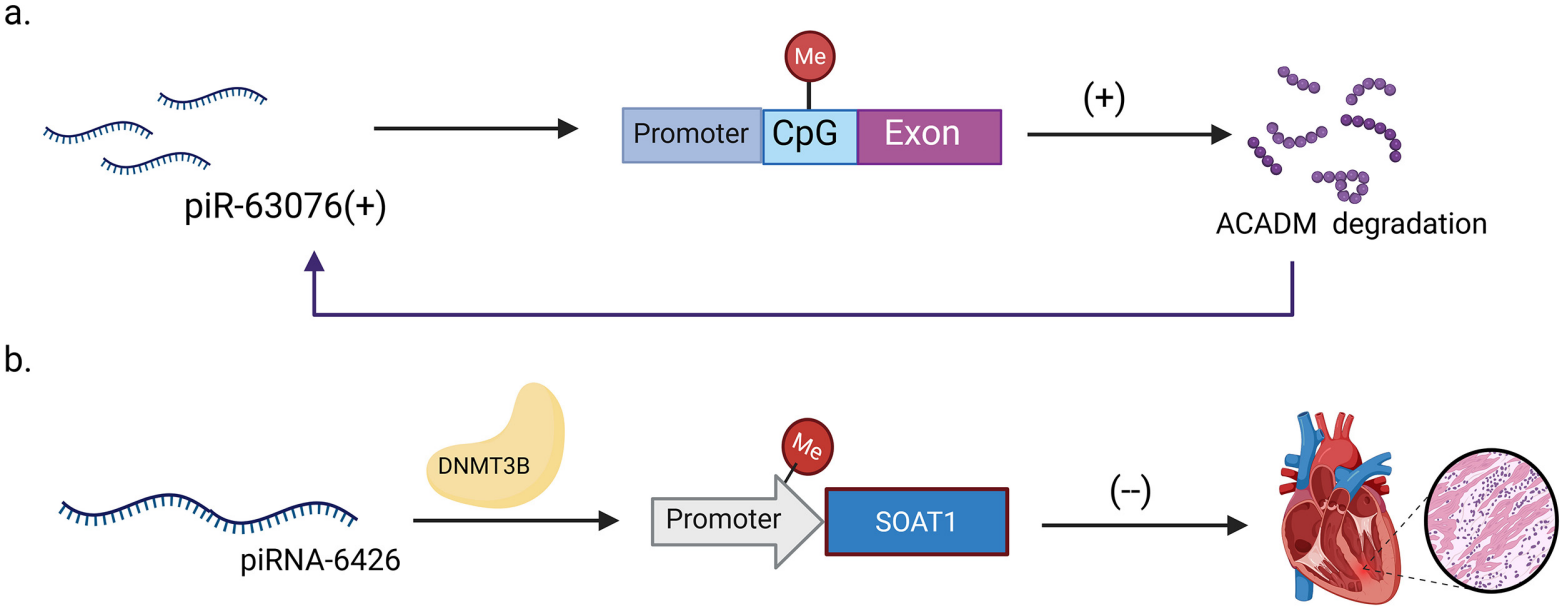
**Mechanistic roles of piRNAs in different cardiovascular diseases (CVDs)**. (a) In pulmonary arterial hypertension, piRNA-63076 has been implicated in hypoxia-driven proliferation of pulmonary arterial smooth muscle cells (PASMCs), potentially through regulation of ACADM. (b) In cardiomyocytes, piRNA-6426 has been reported to attenuate hypoxia-induced dysfunction and heart failure–related phenotypes by facilitating DNMT3B-dependent methylation of *SOAT1*. Created in BioRender (https://www.biorender.com/).

In PAH, it is important to distinguish between discovery-stage biomarker candidates and directly supported mechanistic regulators. While DQ593039 is currently better interpreted as an exploratory extracellular vesicle-associated candidate identified in profiling analyses, more direct mechanistic evidence is available for piR-63076, which has been reported to promote pulmonary arterial smooth muscle cell proliferation. Specifically, increased piR-63076 expression has been linked to altered methylation and transcriptional regulation involving the *Acadm *gene, thereby contributing to pulmonary vascular remodeling in experimental settings (Fig. 3a) [37]. These findings suggest that selected piRNAs may participate in PASMC dysfunction and disease progression, although the mechanistic evidence remains limited to a relatively small number of studies.

#### 3.3.3 Translational Relevance and Critical Appraisal

At present, PAH-associated piRNAs are best viewed as early-stage biomarker candidates and experimental mechanistic regulators. The main limitations include small sample size, heterogeneity in sample type (e.g., extracellular vesicles versus vascular cells), incomplete validation across independent populations, and limited standardization of sequencing and annotation pipelines. As in other somatic tissue studies, the possibility that some reported “piRNAs” may represent other small-RNA fragments or annotation artifacts should also be considered when interpreting current PAH findings.

### 3.4 Heart Failure

#### 3.4.1 Discovery and Validation Evidence

In HF, current piRNA-related evidence includes both circulating/exosomal candidates and cell- or tissue-associated mechanistic regulators. In an exosomal profiling study, RNA sequencing and bioinformatic analysis of serum exosomes from patients with HF and healthy controls identified broad differences in piRNA expression, including 585 upregulated and 4623 downregulated piRNAs in the HF group. Among these, hsa-piR-020009 and hsa-piR-006426 were reported as the most prominently downregulated candidates, suggesting potential value as exploratory circulating biomarkers [50]. However, these findings remain discovery-stage observations and still require validation in larger, clinically stratified cohorts.

#### 3.4.2 Mechanistic Evidence

More direct mechanistic evidence is available for piRNA-6426, which has been reported to be reduced in HF and to improve cardiac function when overexpressed in experimental models [51]. Mechanistically, piRNA-6426 has been linked to DNMT3B-mediated methylation of *SOAT1*, thereby suppressing apoptosis, inflammation, and oxidative stress in cardiomyocytes (Fig. 3b) [51]. In addition, recent work has identified a cardiac-abundant and fibroblast-specific piRNA, CFRPi (piRNA-000691), as a regulator of fibrosis in pressure-overloaded HF. In that study, knockdown of CFRPi reduced cardiac fibrosis and improved cardiac function, whereas mechanistic analysis suggested that CFRPi suppresses APLN and influences downstream PI3K-AKT-mTOR signaling [13].

#### 3.4.3 Translational Relevance and Critical Appraisal

Taken together, HF-associated piRNAs are currently best considered exploratory biomarkers and preclinical therapeutic candidates. Although both exosomal candidates and fibrosis-related functional regulators have been described, the overall evidence base remains limited by small cohort size, incomplete external validation, and variability in sample processing, sequencing, and normalization methods. Furthermore, because these observations are derived from somatic cardiovascular tissues and circulating material, careful annotation remains essential to distinguish bona fide piRNAs from other small-RNA fragments before firm mechanistic or translational conclusions can be drawn. Overall, the current cardiovascular piRNA literature remains promising but largely exploratory, and future progress will depend on larger validation cohorts, methodological standardization, and more rigorous annotation in somatic tissues.

## 4. Future Prospects

Although piRNA research in cardiovascular disease remains at an early stage, several concrete priorities are needed to improve biological confidence and translational relevance. First, methodological standardization is essential. Future studies should adopt more rigorous and transparent small-RNA sequencing pipelines for somatic piRNA analysis, including clearly defined size-selection criteria, stringent mapping and annotation workflows, appropriate exclusion of other small-RNA classes, and, where possible, supporting evidence of PIWI association. This is particularly important in cardiovascular tissues, where some reported “piRNAs” may otherwise represent fragments derived from other small noncoding RNAs rather than bona fide PIWI-interacting RNAs [33,56]. Second, orthogonal validation should become routine. Candidate piRNAs identified by discovery-stage sequencing should be validated using independent methods such as qPCR or digital PCR, with careful primer design, stringent negative controls, external spike-in or internal normalization strategies, and replication in independent sample sets. Without such validation, sequencing-based observations should be regarded as exploratory rather than definitive [57,58,59,60]. Third, future cardiovascular studies should place greater emphasis on multicenter replication and clinically well-defined cohorts. Larger studies with standardized sample collection, preprocessing, and phenotype definition will be necessary to determine whether candidate piRNAs are reproducible across disease stages, tissue sources, and patient populations. This is particularly important for distinguishing broadly associated signals from disease-specific biomarkers with real diagnostic or prognostic value [33]. Finally, therapeutic translation remains promising but technically challenging. Although synthetic piRNA mimics or antagonists may eventually have therapeutic potential, major barriers remain, including tissue-specific delivery, intracellular uptake, off-target effects, immune activation, and long-term safety [61,62]. More realistic preclinical development will require validated disease models, cell-type-specific targeting strategies, and careful comparison of delivery platforms such as engineered extracellular vesicles and lipid-based nanoparticles [63,64].

## 5. Conclusion

In this context, emerging tools such as multi-omics integration, organoid systems, and AI-assisted modeling may help prioritize functionally relevant candidates, but these approaches should complement—rather than replace—rigorous experimental validation and reproducible clinical study design. Overall, progress in the cardiovascular piRNA field will depend less on the identification of additional candidate molecules alone and more on the establishment of robust analytical standards, reproducible validation frameworks, and biologically realistic translational strategies [33].
